# Miconazole Suppresses 27-Hydroxycholesterol-induced Inflammation by Regulating Activation of Monocytic Cells to a Proinflammatory Phenotype

**DOI:** 10.3389/fphar.2021.691019

**Published:** 2021-10-22

**Authors:** Bo-Young Kim, Yonghae Son, Hyok-rae Cho, Dongjun Lee, Seong-Kug Eo, Koanhoi Kim

**Affiliations:** ^1^ Department of Pharmacology, Pusan National University-School of Medicine, Yangsan, Korea; ^2^ Department of Neurosurgery, College of Medicine, Kosin University, Busan, Korea; ^3^ Department of Convergence Medicine, Pusan National University-School of Medicine, Yangsan, Korea; ^4^ College of Veterinary Medicine and Bio-Safety Research Institute, Jeonbuk National University, Iksan, Korea

**Keywords:** 27-hydroxychoelsterol, inflammation, miconazole, CD14, ORP3

## Abstract

Miconazole is effective in treating inflammatory skin conditions and has well-established antifungal effects. To elucidate the underlying mechanisms mediating its additional beneficial effects, we assessed whether miconazole influences the inflammation induced by 27-hydroxycholesterol (27OHChol), an oxygenated cholesterol derivative with high proinflammatory activity, using THP-1 monocytic cells. Miconazole dose-dependently inhibited the expression of proinflammatory markers, including CCL2 and CCR5 ligands such as CCL3 and CCL4, and impaired the migration of monocytic cells and CCR5-positive T cells. In the presence of 27OHChol, miconazole decreased CD14 surface levels and considerably weakened the lipopolysaccharide response. Furthermore, miconazole blocked the release of soluble CD14 and impaired the transcription of the matrix metalloproteinase-9 gene and secretion of its active gene product. Additionally, it downregulated the expression of ORP3 and restored the endocytic function of THP-1 cells. Collectively, these findings indicate that miconazole regulates the 27OHChol-induced expression of proinflammatory molecules in monocytic cells, thereby suppressing inflammation in an oxysterol-rich milieu.

## Introduction

Miconazole, an imidazole antifungal agent, is topically applied to the skin or mucous membranes for treating fungal infections (Barasch and Griffin, 2008; Zhang et al., 2016). The imidazole antifungal agents inhibit the synthesis of ergosterol, the most abundant sterol in the fungal cell membrane, regulate permeability and fluidity, and induce the accumulation of reactive oxygen species within the fungal organism, thereby exerting fungistatic or fungicidal effects (Barasch and Griffin, 2008). Previous studies have revealed the anti-inflammatory activity of miconazole at therapeutic equivalent doses by employing cell and animal models (Zhang et al., 2019; Yeo et al., 2020). Miconazole reportedly reduces neuroinflammation in lipopolysaccharide (LPS)-treated mice by binding to inducible nitric oxide synthase (iNOS), a major downstream mediator of inflammation (Nakazawa et al., 2017). Miconazole also impairs receptor activator of NF-κB ligand-induced expression of the proinflammatory cytokines (Zheng et al., 2014). Furthermore, topical miconazole can effectively treat diaper dermatitis in infants, an acute skin inflammatory reaction in the diaper region (Concannon et al., 2001), as well as ameliorate plaque psoriasis (Niczyporuk and Krajewska-Kulak, 1997). However, underlying mechanisms mediating these anti-inflammatory effects of miconazole remain elusive.

Oxysterols, oxidative derivatives of cholesterol, are formed via enzymatic oxidation or non-enzymatic autoxidation processes (Brown and Jessup, 1999). Among known oxysterols, 27-hydroxycholesterol (27OHChol), followed by 7-oxysterols, is the major oxysterol in circulation (Brown and Jessup, 1999; Garcia-Cruset et al., 2001). 27OHChol is a bioactive lipid involved in lipid metabolism, cell growth, and inflammation. Reportedly, 27OHChol downregulates the expression of sterol regulatory element-binding protein-1 (SREBP-1a) and 3-hydroxy-3-methylglutaryl-CoA reductase (HMG-CR), thereby decreasing cholesterol levels (An et al., 2017). Oxysterol serves as an endogenous selective estrogen receptor modulator (SERM) and promotes the growth of estrogen receptor-positive breast cancer cells (Dusell et al., 2008; Wu et al., 2013). Furthermore, 27OHChol has been shown to enhance inflammatory processes that facilitate atherosclerosis by activating endothelial cells and monocytes/macrophages via nuclear receptors like estrogen receptor alpha or LXR (Umetani et al., 2014; Ma and Nelson, 2019; Kim et al., 2021). It induces the migration of monocytic cells and C-C chemokine receptor 5 (CCR5)-positive T cells, which contribute to Th1 dominance, by enhancing chemokine production (Kim et al., 2014), drives polarization of monocytic cells toward a proinflammatory subset, and effects the monocyte-derived dendritic cell differentiation (Son et al., 2013; Asghari et al., 2019; Lee et al., 2019). Moreover, 27OHChol upregulates pattern recognition receptors (PRRs) such as Toll-like receptor (TLR)-6 and CD14, thereby enhancing the response to FSL-2, a TLR2/6 agonist, and LPS, a TLR4 agonist (Heo et al., 2014; Kim et al., 2015). These reports suggest that 27OHChol influences inflammatory responses via multiple mechanisms.

In the present study, we aimed to clarify the mechanisms underlying the anti-inflammatory effects of miconazole. Accordingly, we examined whether miconazole impairs the inflammatory responses induced by 27OHChol which activates monocytic cells to a proinflammatory phenotype. We evaluated gene expression and demonstrated the suppressive effects of this antifungal agent on the expression of chemokines and molecules involved in inflammation using 27OHChol-activated THP-1 monocytic cells, which indicated its potent anti-inflammatory activity in an oxysterol-rich environment. Additionally, we determined whether miconazole influences immune cell migration and phagocytic function.

## Materials and Methods

### Cell Culture

Human THP-1 monocytic cells were purchased from ATCC (Manassas, VA, United States) and cultured in RPMI medium 1,640, supplemented with 10% fetal bovine serum, at 37°C under a humidified atmosphere of 5% CO_2_. Penicillin and streptomycin were added to prevent bacterial contamination. CCR5-expressing Jurkat T cells were maintained in the presence of geneticin, as previously described (Park et al., 2007).

### Activation of Monocytic Cells to a Proinflammatory Phenotype by Treatment With 27OHChol

THP-1 cells in complete culture medium were harvested, washed twice, resuspended in RPMI medium (2.5 × 10^5^ cells/mL) supplemented with 0.1% BSA (endotoxin-free), and incubated overnight. The serum-starved THP-1 cells were activated to a proinflammatory phenotype by treating with 27OHChol. After incubation for 48 h in the presence or absence of miconazole, the cells were harvested by centrifugation (500 × g, 5 min). Supernatants were collected in fresh tubes and used in ELISA, zymography, and chemotaxis assay, and total RNA isolated from the THP-1 cells were used to assess mRNA expression of the genes. Experiments were performed in triplicate and repeated at least three times.

### Reagents

27OHChol was purchased from Santa Cruz Biotechnology, Inc. (Santa Cruz, CA, United States). Miconazole and LPS were purchased from Sigma-Aldrich (St. Louis, MO, United States). Anti-CD14, anti-ORP3, and anti-β-actin antibodies were purchased from Santa Cruz Biotechnology, Inc.

### Quantitative Reverse Transcription–Polymerase Chain Reaction (qRT-PCR)

Total RNA was purified from THP-1 cells (1 × 10^6^ cells) with TRI reagent (Sigma-Aldrich). After reverse transcription of total RNA at 42°C for 1 h, qRT-PCR was performed in triplicate using a LightCycler 96 Real-time PCR System (Roche, Basel, Switzerland). The thermal cycling conditions were as follows: 95°C for 10 min, followed by 45 cycles at 95°C for 10 s, 50°C for 10 s, and 72°C for 10 s. The relative expression of each gene was calculated as the ratio to the expression of housekeeping gene (*GAPDH*) using LightCycler^®^ 96 (version 1.1.0.1320; Roche). The levels of target gene mRNA were normalized to those of *GAPDH* using the 2^−ΔΔCt^ method (Livak and Schmittgen, 2001). The primers used were as follows: CCL2, 5ʹ-CAG​CCA​GAT​GCA​ATC​AAT​GCC-3ʹ (forward) and 5ʹ-TGG​AAT​CCT​GAA​CCC​ACT​TCT-3ʹ (reverse); CCL3, 5ʹ-AGT​TCT​CTG​CAT​CAC​TTG​CTG-3ʹ (forward) and 5ʹ-CGG​CTT​CGC​TTG​GTT​AGG​AA-3ʹ (reverse); CCL4, 5ʹ-CTG​GGT​CCA​GGA​GTA​CGT​GT-3ʹ (forward) and 5ʹ-GCG​GAG​AGG​AGT​CCT​GAG​TA-3ʹ (reverse); CD14, 5ʹ-ACG​CCA​GAA​CCT​TGT​GAG​C-3ʹ (forward) and 5ʹ-GCA​TGG​ATC​TCC​ACC​TCT​ACT​G-3ʹ (reverse); MMP-9, 5ʹ-GCA​CGA​CGT​CTT​CCA​GTA​CC-3ʹ (forward) and 5ʹ-CAG​GAT​GTC​ATA​GGT​CAC​GTA​GC-3ʹ (reverse); GAPDH, 5′-GAA​GGT​GAA​GGT​CGG​AGT-3’ (forward) and 5′-GAA​GAT​GGT​GAT​GGG​ATT​TC-3’ (reverse).

### Enzyme-Linked Immunosorbent Assay (ELISA)

Cell culture supernatants (10 ml) were collected after treatment of serum-starved THP-1 cells with miconazole and 27OHChol. The protein levels of chemokines, soluble CD14 (sCD14), and matrix metalloproteinase (MMP)-9 secreted into the culture media were measured using commercially available ELISA kits following the manufacturer’s instructions (R&D Systems, Minneapolis, MN, United States).

### Migration Assay

Cell migration was examined using Transwell Permeable Supports with 5 μm-pore polycarbonate Transwell inserts (Costar, Cambridge, MA, United States), as previously described (Kim et al., 2017). The insert chambers loaded with THP-1 cells or CCR5-expressing Jurkat T cells (5 × 10^5^ cells each in 100 μl of 0.1% BSA in RPMI) were inserted into wells filled with supernatants isolated from THP-1 cells treated with 27OHChol in the presence and absence of miconazole. After incubation at 37°C, cells in the bottom chamber were counted using a Vi-Cell XR cell counter (Beckman Coulter, Indianapolis, IN, United States).

### MMP-9 Gelatinolytic Activity

MMP-9 activity was assessed via gelatin zymography, as detailed previously (Kim et al., 2017). Briefly, Cell culture supernatants (4 ml) collected from THP-1 cells (1× 10^6^ cells) grown in serum-free medium were concentrated 30-fold and separated on 8% polyacrylamide gels containing 0.15% gelatin. After staining with 0.2% Coomassie brilliant blue R-250, gels were destained with methanol/acetic acid (20/10%) to visualize the gelatinolytic activity. Gels were photographed using ZoomBrower EX5.0 (Canon, Tokyo, Japan).

### Flow Cytometric Analysis

THP-1 cells (1 × 10^6^ cells) treated with miconazole and 27OHChol were harvested by centrifugation. After incubation with an anti-CD14 antibody conjugated with a fluorescent dye at 4°C for 40 min, cells were washed and resuspended in 1% paraformaldehyde prepared in phosphate-buffered saline. Fluorescence was analyzed with FACSCanto™II using the FACS Diva™ 6.0 software (BD Bioscience, Franklin Lakes, NJ, United States).

### FITC-Dextran Uptake Assay

Following treatment with miconazole and 27OHChol, THP-1 cells (1.0 × 10^6^ cells) were resuspended in a culture medium containing 0.5 mg/ml FITC-conjugated dextran (40 kDa) and incubated at 37°C for 30 min. The resultant fluorescence was analyzed by flow cytometry. Experiments were performed in triplicate and repeated at least three times.

### Western Blot Analysis

THP-1 cells (1 × 10^6^ cells) were lysed with lysis buffer (1% SDS, 1 mM NaVO3 and 10 mM Tris-HCl, pH 7.4) containing protease inhibitors, and the supernatants were isolated following centrifugation (15,000 × g) for 5 min at 4°C. Cell lysates were separated via 10% sodium dodecyl sulfate-polyacrylamide gel electrophoresis and transferred onto nitrocellulose membranes. After blocking for 1 h in 1% skim milk (in Tris-buffered saline (TBS) containing 0.05% Tween-20), membranes were incubated with primary antibodies, diluted in the blocking solution, overnight at 4°C. Then, the membranes were washed with TBS and incubated with horseradish peroxidase (HRP)-conjugated secondary antibodies at room temperature for 1 h. After washing, bands were detected using chemiluminescent reagents. Chemiluminescence images were captured using Amersham Imager 600 (GE Healthcare Life Sciences, Pittsburgh, PA, United States).

### Statistical Analysis

Statistical analysis was performed using one-way ANOVA, followed by Dunnett’s multiple comparison test, using GraphPad Prism (GraphPad Software Inc, San Diego, CA, United States). Statistical significance was set at *p < 0.05*.

## Results

### Miconazole Suppresses the Chemokine Expression and Immune Cell Migration Induced by 27OHChol

**FIGURE 1:**
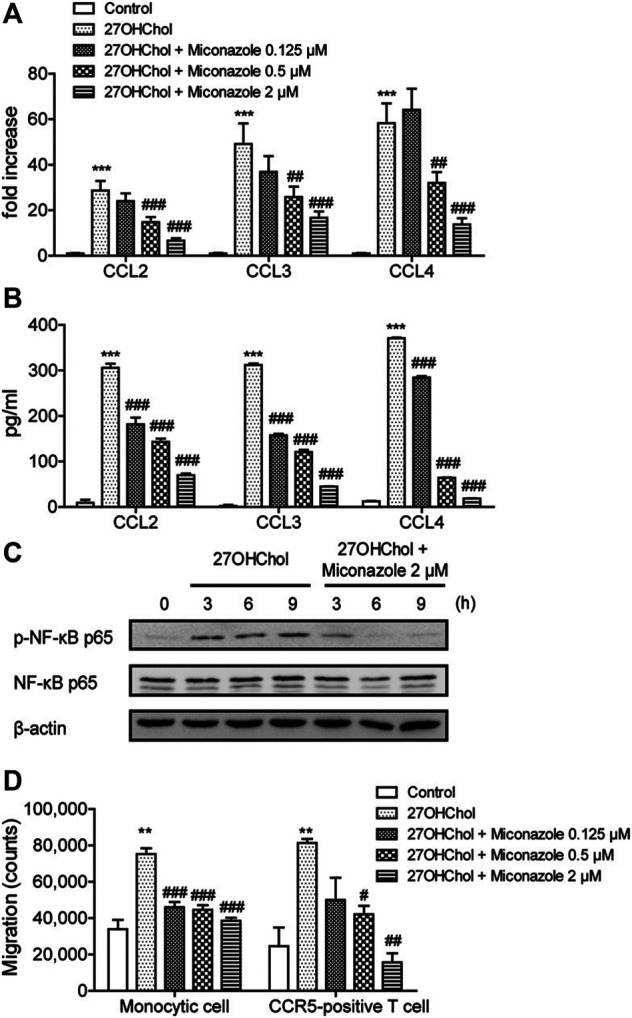
Miconazole downregulates chemokine expression and p65 phosphorylation and decreases immune cell migration. THP-1 cells (2.5 × 10^5^ cells/ml) were serum-starved by overnight incubation in RPMI medium supplemented with 0.1% bovine serum albumin (BSA; endotoxin-free), and cultured for 48 h with 27OHChol (2 μg/ml) in the presence of indicated miconazole concentrations. **(A)** The transcript levels of CCL2, CCL3, and CCL4 were assessed using quantitative reverse transcription-PCR. The *y*-axis values represent the increase in the mRNA levels of indicated genes normalized to GAPDH levels relative to those of the non-treated THP-1 cells (control). **(B)** Protein levels of CCL2, CCL3, or CCL4 in the media were measured with ELISA. Results are representative of three independent experiments. Data are expressed as mean ± standard deviation (SD) (*n* = 3 replicates for each group). ***p < 0.001 *vs* control; ###*p* < 0.001 *vs* 27OHChol; ##*p* < 0.01 *vs* 27OHChol. **(C)** Monocytic cells and Jurkat T cells expressing CCR5 were exposed to isolated conditioned media, and cell migration was measured using a chemotaxis assay. Results are representative of three independent experiments. Data are expressed as mean ± SD (*n* = 3 replicates for each group). ***p < 0.001 *vs* control; ###*p* < 0.001 *vs* 27OHChol; ##*p* < 0.01 *vs* 27OHChol; #*p* < 0.05 *vs* 27OHChol. **(D)** Serum-starved THP-1 cells were exposed to 27OHChol (2 μg/ml) for indicated periods in the absence or presence of miconazole (2 μM). Isolated cell extracts were subjected to immunoblotting for β-actin and the phosphorylated and unphosphorylated forms of p65. Immunoblots are representative of three independent experiments. 27OHChol, 27-hydroxycholesterol; CCR5, C-C chemokine receptor 5.

To determine whether miconazole affects inflammatory responses, we examined its effects on 27OHChol-induced chemokine expression. Herein, 27OHChol-induced stimulation of THP-1 cells upregulated the expression of CCL2, CCL3, and CCL4 chemokines at both mRNA and protein levels; however, this increase was suppressed by miconazole, as determined with qRT-PCR and ELISA (Figure 1A,B). Notably, the transcript levels of CCL2, CCL3, and CCL4 were significantly reduced following treatment with 0.5 and 2 μM miconazole (*p* < 0.0001), while secretion of CCL2, CCL3, and CCL4 was dose-dependently inhibited (*p* < 0.0001). Furthermore, we assessed whether miconazole affected the expression of proinflammatory and anti-inflammatory markers and found that the antifungal agent suppressed the 27OHChol-induced expression of proinflammatory markers, including interleukin (IL)-1β, tumor necrosis factor (TNF)-α, C-X-C motif chemokine ligand 10 (CXCL10), CXCL11, CD80, and CD86, as well as anti-inflammatory markers, like CD163 and CD206 (Supplementary Figure S1). Transcripts of IL-6 or IL-10 genes were undetectable in the presence or absence of 27OHChol and miconazole, as detected by RT-PCR (data not shown). These results indicated that miconazole significantly impaired expression of proinflammatory markers when compared with anti-inflammatory molecules.

The expression of chemokines is regulated by nuclear factor kappa B (NF-κB), and phosphorylation of the p65 subunit activates the selective transcription of downstream proinflammatory genes (Shi and Berger, 2018). Therefore, we investigated the effects of miconazole on p65 phosphorylation. Increased p65 phosphorylation was detected at 3, 6, and 9 h following treatment with 27OHChol; however, this increased phosphorylation was suppressed in the presence of miconazole (Figure 1C). These results indicated that miconazole might regulate the expression of inflammatory genes induced by 27OHChol by modulating p65 phosphorylation.

In addition, chemotaxis assays were performed to evaluate whether miconazole influences the migration of inflammatory immune cells such as monocytes/macrophages and T cells. Migration of THP-1 monocytic cells was significantly increased in response to treatment with the supernatant isolated following 27OHChol-induced stimulation. However, such a migration was inhibited when cells were exposed to supernatants harvested after 27OHChol stimulation with miconazole treatment at final concentrations of 0.125, 0.5, or 2 μM (*p* < 0.0001, Figure 1D). Furthermore, CCR5-positive T cells migrated in response to the supernatant isolated following 27OHChol-induced stimulation; however, cell migration was reduced when supernatants were harvested in the presence of 27OHChol plus miconazole at a final concentration of 0.5 or 2 μM (*p* = 0.0012, Figure 1D). Collectively, these results indicate that miconazole inhibits the expression of inflammatory chemokines and, thereby, immune cell migration induced by 27OHChol.

### Miconazole Downregulates CD14 Expression and Weakens LPS Response

**FIGURE 2:**
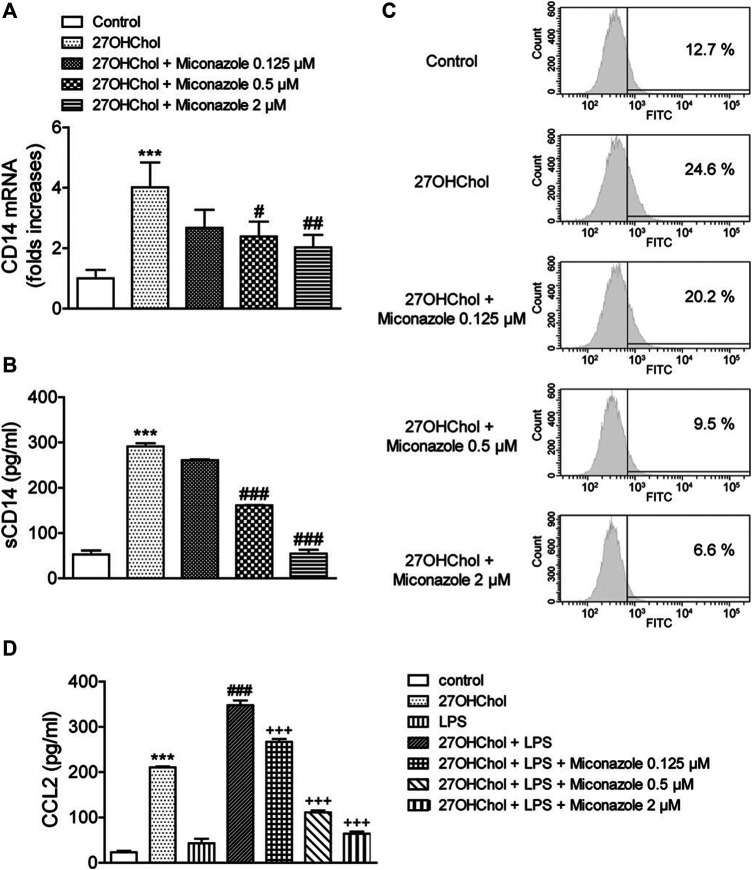
Miconazole downregulates CD14 expression, thereby weakening the LPS response. THP-1 cells (2.5 × 10^5^ cells/ml) were cultured for 48 h with 27OHChol (2 μg/ml) in the presence of indicated miconazole concentrations. **(A)** CD14 transcript levels were assessed *via* quantitative reverse transcription-PCR, and **(B)** the amount of sCD14 protein secreted into the medium was measured with ELISA. Results are representative of three independent experiments. Data are expressed as mean ± standard deviation (SD) (*n* = 3 replicates for each group). ***p < 0.001 *vs* control; ###*p* < 0.001 *vs* 27OHChol; ##*p* < 0.01 *vs* 27OHChol; #*p* < 0.05 *vs* 27OHChol. **(C)** THP-1 cells immunostained with surface CD14 were analyzed via flow cytometry. Data are representative of three independent experiments. **(D)** Serum-starved THP-1 cells (2.5 × 10^5^ cells/ml) were cultured for 24 h with 27OHChol (2 μg/ml) in the presence of the indicated concentrations of miconazole, followed by stimulation for 9 h with or without LPS (100 ng/ml) obtained from *Escherichia coli* K12. Culture media were isolated, and the amount of CCL2 protein secreted into the media was measured using ELISA. Results are representative of three independent experiments. Data are expressed as mean ± SD (*n* = 3 replicates for each group). ***p < 0.001 *vs* control; ###*p* < 0.001 *vs* 27OHChol; +++ *p* < 0.001 *vs* 27OHChol plus LPS. 27OHChol, 27-hydroxycholesterol; LPS, lipopolysaccharide; sCD14, soluble CD14.

We next determined whether miconazole affects innate immune responses, by examining its effects on CD14 expression and response to LPS. Stimulation of THP-1 cells with 27OHChol resulted in increased CD14 transcript levels; however, this increase was suppressed in the presence of 0.5 or 2 μM miconazole (*p* = 0.0009, Figure 2A). Additionally, the 27OHChol-stimulated THP-1 cells released sCD14, which was suppressed following exposure to 0.5 or 2 μM miconazole (*p* < 0.0001, Figure 2B). Notably, the amount of sCD14 released was reduced to basal levels in the presence of 2 μM miconazole. We then evaluated whether miconazole altered the levels of membrane-bound CD14 (mCD14). We observed that 27OHChol-induced stimulation increased the percentage of CD14-positive cells, indicating the upregulation of CD14 expression on the cell surface; however, this percentage decreased in the presence of miconazole in a dose-dependent manner. Treatment with 0.5 or 2 μM miconazole profoundly reduced the mCD14 levels on 27OHChol-stimulated THP-1 cells when compared with those on control cells (Figure 2C). These results indicate that miconazole downregulates CD14 expression.

Notably, mCD14 is involved in LPS-induced monocyte/macrophage activation. Accordingly, we investigated whether miconazole regulates LPS responses, by measuring CCL2 expression levels (Figure 2D). The addition of LPS to 27OHChol-exposed THP-1 cells further enhanced CCL2 secretion when compared with that observed following stimulation with 27OHChol or LPS alone, i.e., CCL2 secretion was enhanced by 27OHChol plus LPS; however, this secretion was significantly inhibited by miconazole (*p* < 0.0001). Moreover, miconazole significantly decreased the transcript levels of CCL2 induced by 27OHChol plus LPS, but not by LPS alone, as determined with qRT-PCR (Supplementary Figure S2). Collectively, these results suggest that miconazole regulates 27OHChol-enhanced LPS responses by downregulating CD14 expression.

### Miconazole Inhibits MMP-9 Activity

**FIGURE 3:**
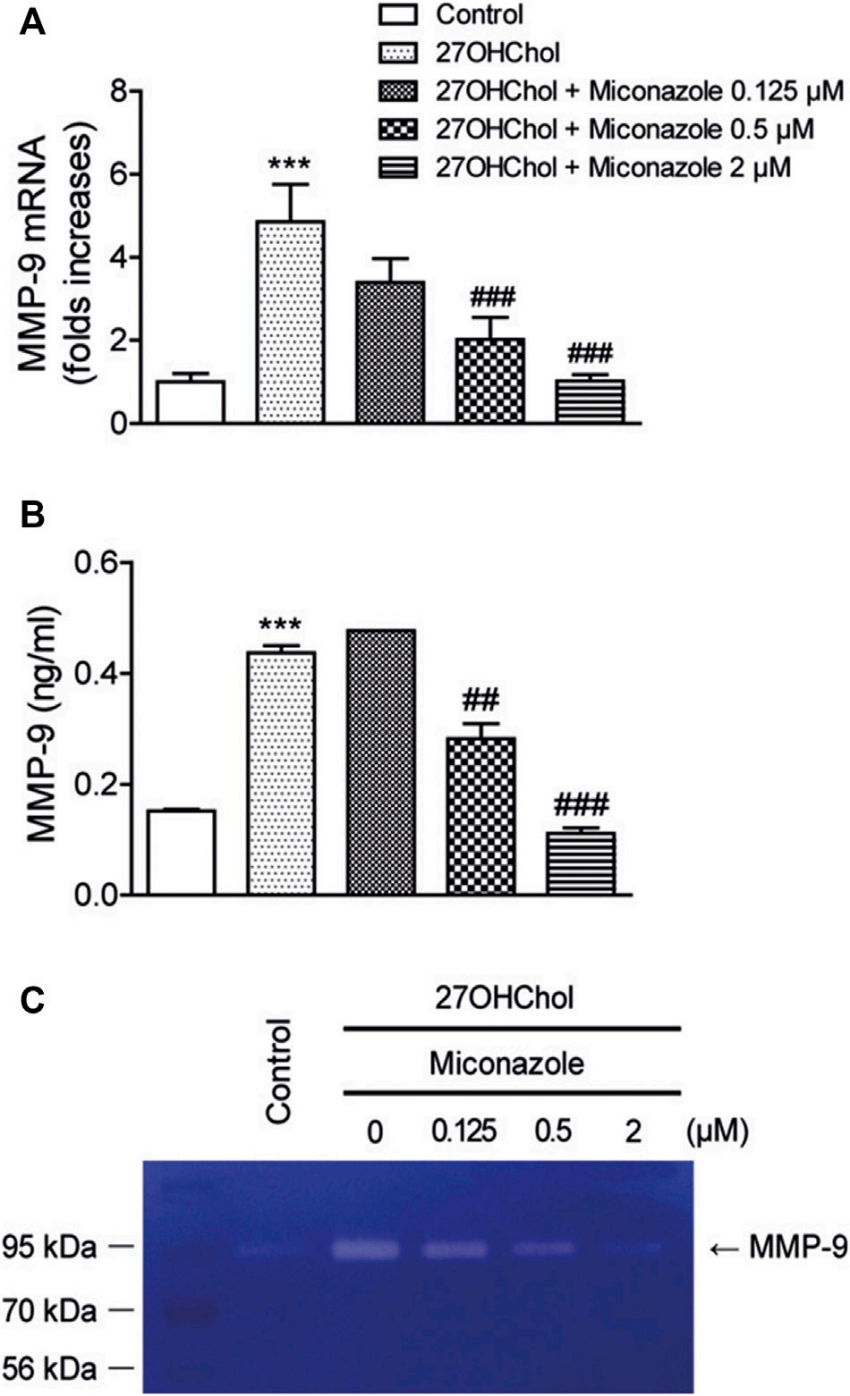
Miconazole downregulates MMP-9 activity via transcriptional regulation. Serum-starved THP-1 cells were cultured for 48 h with 27OHChol in the presence of indicated miconazole concentrations. **(A)** MMP-9 transcript levels were assessed *via* quantitative reverse transcription-PCR, and **(B)** the amount of MMP-9 secreted into the medium was measured with ELISA. Results are representative of three independent experiments. Data are expressed as mean ± standard deviation (SD) (*n* = 3 replicates for each group). ***p < 0.001 *vs* control; ###*p* < 0.001 *vs* 27OHChol; ##*p* < 0.01 *vs* 27OHChol. **(C)** The activity of MMP-9 secreted by THP-1 cells was assessed via gelatin zymography. Control cells were cultured for 48 h in medium alone. The image is representative of three independent experiments. 27OHChol, 27-hydroxycholesterol; MMP-9, matrix metalloproteinase-9.

MMP-9 is involved in macrophage infiltration and the cleavage of mCD14 to sCD14. Accordingly, we investigated the effects of miconazole on MMP-9 expression by evaluating MMP-9 transcript and protein levels. Exposure of THP-1 monocytic cells to 27OHChol resulted in enhanced transcription of MMP-9 and secretion of its gene product, which was significantly suppressed in the presence of 0.5 or 2 μM miconazole, as determined with qRT-PCR (*p* < 0.0001) and ELISA (*p* < 0.0001, Figures 3A,B). Furthermore, on evaluating the effects of miconazole on MMP-9 activity, we observed that the gelatinolytic activity in the cell supernatant was increased following exposure to 27OHChol; this elevated activity was suppressed by miconazole treatment, as revealed by zymography results (Figure 3C). These findings indicated that miconazole suppresses the MMP-9 activity enhanced by 27OHChol.

### Miconazole Restores Endocytic Activity

**FIGURE 4:**
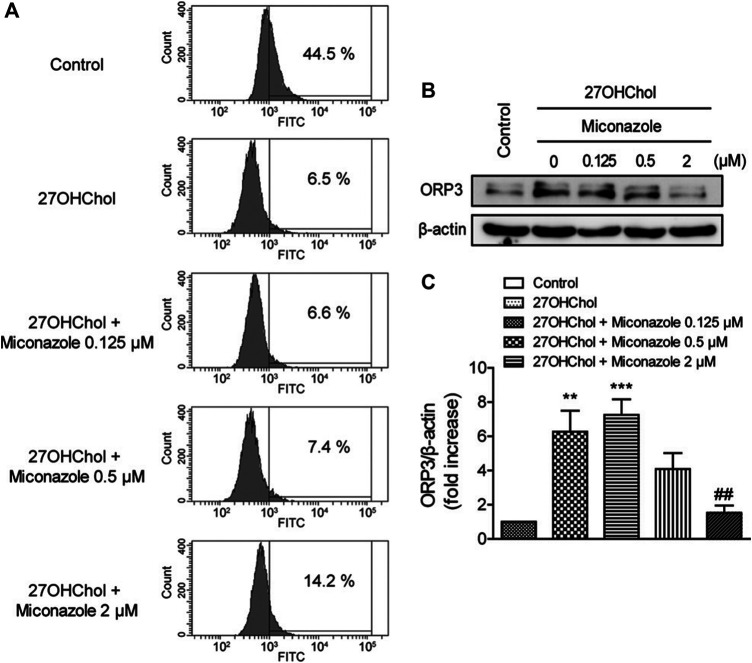
Miconazole partially restores the endocytic function of monocytic cells. Serum-starved THP-1 cells were cultured for 48 h with 27OHChol in the presence of indicated miconazole concentrations. **(A)** THP-1 cells were exposed to 1 mg/ml FITC-conjugated dextran for 1 h, and the percentage of cells exhibiting high fluorescence intensity was determined using flow cytometry. Data are representative of three independent experiments. **(B)** Isolated cell extracts were subjected to immunoblotting for β-actin and ORP3. Immunoblots are representative of three independent experiments. **(C)** ORP protein levels were quantified using densitometry. Data are expressed as the mean ± standard error (SE) of values from three independent experiments. ***p < 0.001 *vs* control; **p < 0.01 *vs* control; ##*p* < 0.01 vs 27OHChol. 27OHChol, 27-hydroxycholesterol.

We examined whether miconazole affects the endocytic function of THP-1 cells by assessing its effects on endocytosis. Following 27OHChol stimulation, the percentage of cells exhibiting endocytic activity decreased from 44.5 to 6.5%. Nonetheless, treatment with miconazole restored endocytic function; the percentage of cells displaying endocytic activity increased to 14.2% in the presence of 2 μM miconazole (Figure 4A). Furthermore, we assessed the expression of OSBP-related protein 3 (ORP3), which regulates the phagocytic function. Compared with those in control cells, the expression levels of ORP3 were increased in 27OHChol-stimulated THP-1 cells; however, this increase was suppressed in the presence of 2 μM miconazole (*p* < 0.0001, Figures 4B,C). These results suggest that miconazole alters the endocytic ability of monocytic cells and suppresses the expression of ORP3.

## Discussion

Previous reports indicate that 27OHChol is an endogenous immunosterol involved in inflammation and immune response by effecting activation and differentiation of monocytic cells into a proinflammatory subset and a phenotypically mature dendritic cell type, respectively (Kim et al., 2013; Son et al., 2013; Heo et al., 2014; Kim et al., 2014; Kim et al., 2015). Therefore, drugs that regulate the activities of 27OHChol are likely to have anti-inflammatory or immunosuppressive effects. Accordingly, immunosuppressive drugs, such as dexamethasone, prednisolone, and cyclosporine A, have been reported to inhibit the 27OHChol-induced activation of monocytes (Kim et al., 2017; Lee et al., 2019; Kim et al., 2020). In addition, diclofenac, a non-steroidal anti-inflammatory drug, and reblastatin, which exhibits anticancer effects, and 4-*O*-methylalpinumisoflavone, which displays anti-inflammatory effects against LPS, suppress 27OHChol-induced monocytic cell activation (Son et al., 2017; Lee et al., 2019; Choi et al., 2020). Herein, we revealed a novel pharmacological activity of miconazole, the suppression of 27OHChol-induced inflammation, which possibly contributes to its anti-inflammatory effects. We believe that the suppressive effects of miconazole are specific and cannot be attributed to cytotoxicity, as cell death was not detected at treatment concentrations and duration employed in the present study (Supplementary Figure S3). Furthermore, we assessed whether other imidazole antifungal agents could impact the 27OHChol-induced expression of CCL2. The expression of CCL2 was suppressed by econazole and sulconazole, but not by ketoconazole (Supplementary Figure S4). These findings suggest that imidazole antifungal agents have distinct effects on the 27OHChol-induced gene expression of monocytic cells.

Damage-associated molecular patterns (DAMPs) are intracellular molecules that promote the innate immune response (Roh and Sohn, 2018). 27OHChol, which is formed during cholesterol metabolism, activates monocytes/macrophages, thereby stimulating inflammatory responses (Kim et al., 2013; Umetani et al., 2014; Lee et al., 2019). Inflammation is a key aspect of the innate immune response; previous reports suggest that endogenously accumulated 27OHChol can function as a metabolic DAMP. We demonstrated that miconazole suppressed the 27OHChol-induced CCL2 production and monocytic cell migration. As circulating immune cells, including monocytes, migrate into sites of inflammation during the inflammatory response and CCL2 is the key chemoattractant orchestrating migration (Deshmane et al., 2009), our results, along with previous findings, imply that miconazole mediates its therapeutic effects, apart from its antifungal efficacy, by suppressing the inflammation induced by DAMPs which can be released from damaged cells after fungal infections.

As a ubiquitous PRR, CD14 mediates the binding of different bacterial components that function as pathogen-associated molecular patterns (PAMPs) and promotes cell activation (Wu et al., 2019). CD14 binds LPS from Gram-negative bacteria and mediates the LPS response through TLR4, and sCD14 enables the response of endothelial cells, which lack mCD14, to LPS (Pugin et al., 1993; Ciesielska et al., 2021). In addition, CD14 binds to peptidoglycan and lipoteichoic acid, the cell wall components of Gram-positive bacteria, and enhances soluble peptidoglycan signaling through TLR2 (Schwandner et al., 1999). Here, treatment with miconazole downregulated CD14 expression and suppressed the 27OHChol-enhanced LPS response, suggesting that this antifungal agent can modify the CD14-mediated responses to PAMPs. Moreover, CD14 interacts with S100A9, a calcium-binding protein that acts as a DAMP, and this interaction is essential for the S100A9-mediated inflammatory response (He et al., 2016). Collectively, our results support the postulation that miconazole downregulates CD14 expression and, thereby, influences the inflammatory response in a 27OHChol-rich milieu. Accordingly, we propose that miconazole may suppress the inflammatory responses induced by certain PAMPs or DAMPs by regulating PRRs.

ORP3 interacts with the small GTPase R-Ras, which regulates cell adhesion, spreading, and migration. Reportedly, ORP3 overexpression decreases the phagocytic uptake of latex beads in macrophages (Lehto et al., 2008). We demonstrated that the increased ORP3 expression levels overlapped with increased cell migration and decreased phagocytic function after 27OHChol-induced stimulation. Treatment with miconazole reduced ORP3 expression to a basal level, accompanied by impaired cell migration and restored phagocytic activity. These findings suggest a possibility that 27OHChol and miconazole may exert their effects via the R-Ras signaling pathway.

It has been suggested that miconazole may represent a potential chemotherapeutic agent for specific cancers, as it inhibits the expression of gene products in cancer cells (Bruserud, 2001; Mun et al., 2004; Yuan et al., 2017). Using a human acute monocytic leukemia cell line, we revealed that miconazole impairs the expression of chemokines and MMP-9 induced by 27OHChol, which promotes the proliferation of breast and prostate cancer cells (Marwarha et al., 2017). Chemokines affect the proliferation and migration of cancer cells. For example, CCL2, expressed by cancer and stromal cells in the tumor microenvironment, induces tumor cell proliferation at the primary tumor site and stimulates tumor cell migration and invasion into the surrounding extracellular matrix (Hao et al., 2020; Korbecki et al., 2020). In skin cancer, MMP-9, which is secreted from inflammatory cells, degrades the basement membrane and leads to tumor invasion (Pittayapruek et al., 2016). Furthermore, MMP-9 is frequently involved in the development of melanoma and promotes inflammation, angiogenesis, and invasion of tumor keratinocytes (Maru et al., 2014; Napoli et al., 2020). Therefore, the inhibition of MMP-9, along with reduced chemokine production, may contribute to the anticancer effects of miconazole.

In the present study, we reported that miconazole regulates expression of 27OHChol-induced proinflammatory molecules, thus suggesting its potential therapeutic application in disease pathogenesis mediated by 27OHChol. However, the molecular mechanisms underlying this regulation remain unclear. Previous studies have indicated the involvement of multiple signaling pathways. Miconazole suppresses the expression of HIF1α through post-transcriptional regulation in glioma and breast cancer-derived cell lines; this was attributed to the inhibition of mammalian target of rapamycin (mTOR) and regulation of protein synthesis via induction of the phosphorylation of eIF2α, a downstream target of mTOR (Park et al., 2014). In lung cancer cells, miconazole suppresses pSTAT3^Y705^ and inhibits the expression of target genes involved in growth, migration, and invasion (Yoon et al., 2020). Further investigations are needed to determine whether miconazole suppresses the 27OHChol-induced cellular responses by acting on the R-Ras, mTOR, or STAT3 pathway.

## Data Availability

The original contributions presented in the study are included in the article/Supplementary Material, further inquiries can be directed to the corresponding authors
