# Supplementary material for: Miconazole Suppresses 27-Hydroxycholesterol-induced Inflammation by Regulating Activation of Monocytic Cells to a Proinflammatory Phenotype
Source: Front Pharmacol. 2021 Oct 22;12:691019. doi: 10.3389/fphar.2021.691019 (PMC8570190; doi:10.3389/fphar.2021.691019)
Supplement: Supplementary file 1 [file DataSheet1.docx]

Supplementary data

Materials and methods

Quantitative real-time PCR primer sequences for proinflammatory or anti-inflammatory markers evaluated in this study were as follows: CCL2, 5ʹ-CAGCCAGATGCAATCAATGCC-3ʹ (forward) and 5ʹ-TGGAATCCTGAACCCACTTCT-3ʹ (reverse); CXCL10, 5’-TGTACGCTGTACCTGCATCA-3’ (forward) and 5’-GGACAAAATTGGCTTGCAGGA-3’ (reverse); CXCL11, 5’-AAGCAGTGAAAGTGGCAGAT-3’ (forward) and 5’-TAAGCCTTGCTTGCTTCGAT-3’ (reverse); IL-1β, 5’-TGAGCTCGCCAGTGAAATGA-3’ (forward) and 5’-AGATTCGTAGCTGGATGCCG-3’ (reverse); TNF-α, 5’-CCCAGGGACCTCTCT CTAATC-3’ (forward) and 5’-ATGGGCTACAGGCTTGTCACT-3’(reverse); CD80, 5’-GCAGGGAACATCACCATCCA-3’ (forward) and 5’-TCACGTGGATAACACCTGAACA-3’ (reverse); CD86, 5’-GGACTAGCACAGACACACGGA-3’ (forward) and 5’-CTTCAGAGGAGCAGCACCAGA-3’ (reverse); CD163, 5’-AAAAAGCCACAACAGGTCGC-3’ (forward) and 5’-CTTGAGGAAACTGCAAGCCG-3’ (reverse); CD206, 5’-TGAATTGTACTGGTCTGTCCT-3’ (forward) and 5’-CTGTGGTGCTGTGCATTTATCT-3’ (reverse); GAPDH, 5’-GAAGGTGAAGGTCGGAGT-3’ (forward) and 5’-GAAGATGGTGATGGGATTTC-3’ (reverse).


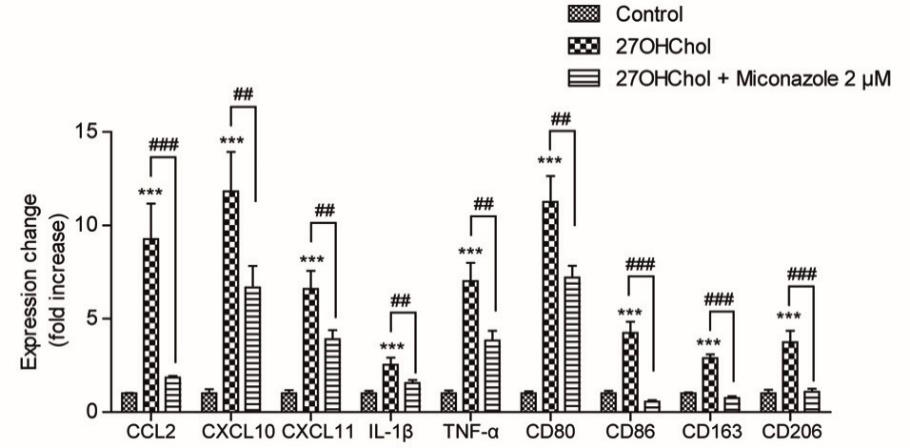


Supplementary Fig. 1. Impaired expression of proinflmmatory/anti-inflammatory polarization markers following miconazole treatment.

THP-1 cells (2.5 × 10^5^ cells/ml) were serum-starved by overnight incubation in RPMI medium supplemented with 0.1 % BSA (endotoxin-free) and cultured for 48 h with 27OHChol (2 μg/ml) in the presence of 2 μM miconazole. The transcript levels of indicated proinflmmatory and anti-inflammatory markers were assessed using quantitative reverse transcription PCR. Experiments were performed in triplicate and repeated three times. Results are representative of the three independent experiments, and data are expressed as mean ± standard deviation (SD) (n = 3 replicates for each group). ***P<0.001, vs. control; ###P<0.001, vs. 27OHChol; ##P<0.01, vs. 27OHChol. 27OHChol, 27-hydroxycholesterol.


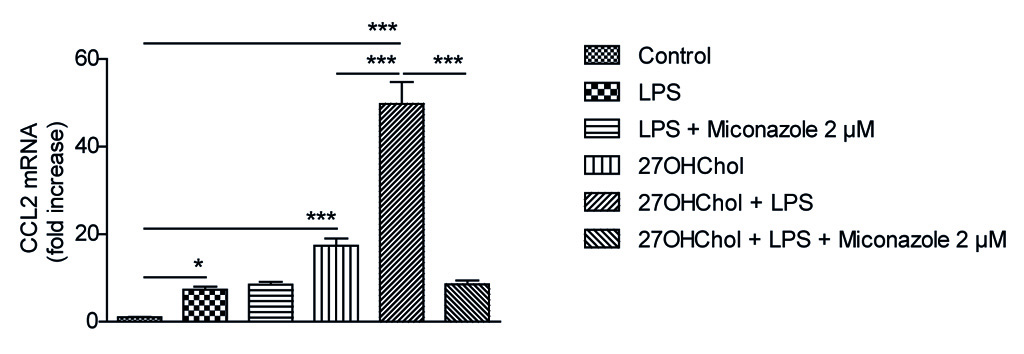


Supplementary Fig. 2. Effects of miconazole treatment on CCL2 expression induced by LPS plus 27OHChol.

THP-1 cells (2.5 × 10^5^ cells/ml) were serum-starved by overnight incubation in RPMI medium supplemented with 0.1 % BSA (endotoxin-free) and treated with 27OHChol (2 μg/ml) for 24 h in the absence or presence of miconazole (2 μM), followed by treatment with LPS (100 ng/ml) for 9 h. CCL2 transcript levels were assessed using quantitative reverse transcription PCR. Experiments were performed in triplicate and repeated three times. Results are representative of the three independent experiments, and data are expressed as mean ± standard deviation (SD) (n = 3 replicates for each group). *** P < 0.001; * P < 0.05. 27OHChol, 27-hydroxycholesterol.


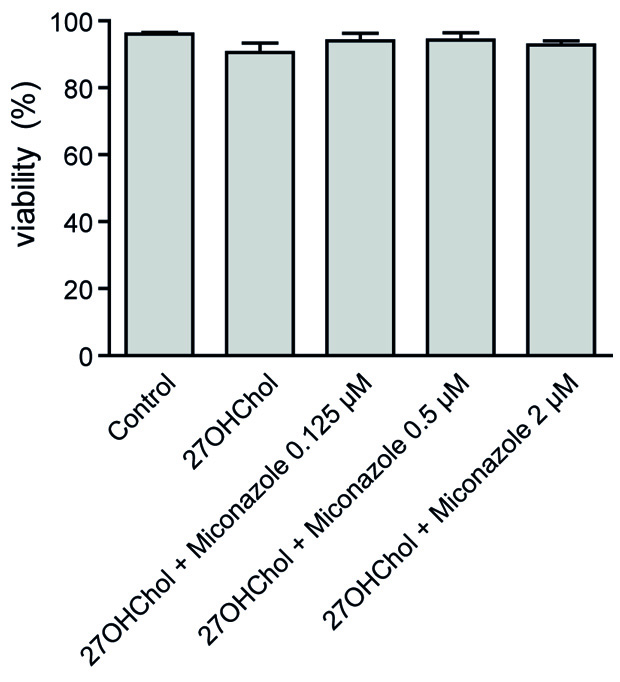


Supplementary Fig. 3. Cell viability following treatment with miconazole.

THP-1 cells (2.5 × 10^5^ cells/ml) were serum-starved by overnight incubation in RPMI medium supplemented with 0.1 % BSA (endotoxin-free) and cultured for 48 h with 27OHChol (2 μg/ml) in the presence of indicated concentrations of miconazole. Cell viability was determined using a Vi-Cell cell counter (Beckman Coulter). The viability of THP-1 cells cultured in medium alone was considered 100 %. Experiments were performed in triplicate and repeated three times. Results are representative of the three independent experiments. Data are expressed as mean ± standard deviation (SD) (n = 3 replicates for each group). 27OHChol, 27-hydroxycholesterol.


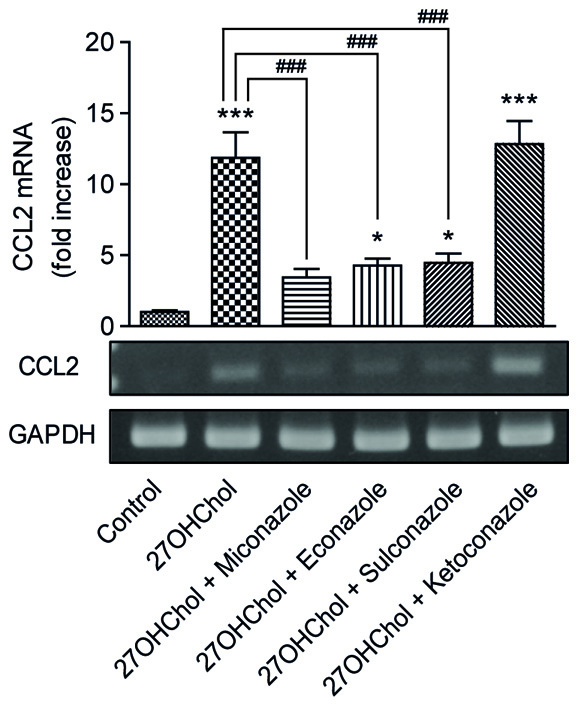


Supplementary Fig. 4. Effects of imidazole antifungal agents on 27OHChol-induced expression of CCL2.

Serum-starved THP-1 cells were cultured for 48 h with 27OHChol (2 μg/ml) in the presence of indicated imidazole antifungal agents (2 μM each), after which the transcripts of the CCL2 gene were evaluated and amplified by quantitative and non-quantitative reverse transcription PCR. Experiments were performed in triplicate and repeated three times. Results are representative of the three independent experiments, and data of quantitative reverse transcription PCR are expressed as mean ± standard deviation (SD) (n = 3 replicates for each group). The PCR bands are representative of three independent experiments. *** P < 0.001 *vs*. control; * P < 0.05 *vs*. control; ### P < 0.001 vs. 27OHChol. 27OHChol, 27-hydroxycholesterol.
